# Current Molecular Targeted Therapies for Bone and Soft Tissue Sarcomas

**DOI:** 10.3390/ijms19030739

**Published:** 2018-03-05

**Authors:** Kenji Nakano, Shunji Takahashi

**Affiliations:** Department of Medical Oncology, Cancer Institute Hospital of Japanese Foundation for Cancer Research, 3-8-31 Ariake, Koto, Tokyo 135-8550, Japan; kenji.nakano@jfcr.or.jp

**Keywords:** soft tissue sarcoma, GIST, bone sarcoma, imatinib, sunitinib, regorafenib, pazopanib, olaratumab, denosumab, immunotherapy

## Abstract

Systemic treatment options for bone and soft tissue sarcomas remained unchanged until the 2000s. These cancers presented challenges in new drug development partly because of their rarity and heterogeneity. Many new molecular targeting drugs have been tried in the 2010s, and some were approved for bone and soft tissue sarcoma. As one of the first molecular targeted drugs approved for solid malignant tumors, imatinib’s approval as a treatment for gastrointestinal stromal tumors (GISTs) has been a great achievement. Following imatinib, other tyrosine kinase inhibitors (TKIs) have been approved for GISTs such as sunitinib and regorafenib, and pazopanib was approved for non-GIST soft tissue sarcomas. Olaratumab, the monoclonal antibody that targets platelet-derived growth factor receptor (PDGFR)-α, was shown to extend the overall survival of soft tissue sarcoma patients and was approved in 2016 in the U.S. as a breakthrough therapy. For bone tumors, new drugs are limited to denosumab, a receptor activator of nuclear factor κB ligand (RANKL) inhibitor, for treating giant cell tumors of bone. In this review, we explain and summarize the current molecular targeting therapies approved and in development for bone and soft tissue sarcomas.

## 1. Introduction

Bone and soft tissue sarcomas are malignant diseases that originate from mesenchymal tissues, regardless of organs. They comprise only approximately 1% of all malignant diseases, but they present more than 50 diagnoses, with heterogenic features in terms of both pathologies and clinical courses [1].

Most bone sarcomas and about half of soft tissue sarcomas originate from extremities (arms and legs) and trunk area surgically treated by orthopedists; however, bone and soft tissue sarcomas also arise from head and neck, gastrointestinal tracts and retroperitoneum, urological and gynecological organs. As for pathological diagnoses, sarcomas are broadly divided into two types; small round cell sarcoma and non-small round cell sarcoma. The former, such as Ewing sarcoma and rhabdomyosarcoma, occurs mainly in young patients and is known to be sensitive to cytotoxic chemotherapies; the latter, on the other hand, is mainly observed in adult patients, and resistant to cytotoxic chemotherapies. Liposarcoma, leiomyosarcoma, synovial sarcoma, and angiosarcoma are representative non-small round cell sarcomas.

Because of the rarity and diversity of bone and soft tissue sarcomas, multidisciplinary approaches are recommended for their treatment [2,3]. Systemic chemotherapies have had an important role in the multidisciplinary care of bone and tissue sarcomas, but for a long time, cytotoxic agents such as doxorubicin were the mainstream systemic therapy for bone and soft tissue sarcomas, except for gastrointestinal stromal tumors (GISTs) [4].

These days, however, investigations of sarcoma genomics and molecular targeted therapy development have brought about a new era of drug treatments for bone and soft tissue sarcoma [5]. The concept of precision medicine and new clinical trial designs have also enabled bone and soft tissue sarcoma patients to participate in individualized clinical trials based on their peculiar mutations [6,7]. Updates of cancer registries and patient consolidation have also contributed to clinical trials [8].

In this review, we provide an overview of the development of molecular targeted therapies for bone and soft tissue sarcomas and the potential targets and challenges.

## 2. Molecular Targeting Therapy for GISTs

### 2.1. Imatinib for GISTs: A Pioneer Targeting Therapy for Sarcomas

GIST account for approx. 8% of soft tissue sarcomas, and they arise from the gastrointestinal submucous tissues, mostly from the submucosa of the stomach [9]. Before the development of molecular targeting drugs, GIST was known to have a poorer prognosis than other soft tissue sarcomas, and to be resistant to cytotoxic chemotherapies; overall survival (OS) of recurrent/metastatic GIST patients were less than one year at that time [10,11].

In the late 1990s, a c-kit (CD117) mutation was identified as a characteristic of GIST, and c-kit was focused on as the target of new drugs [12,13]. Imatinib, a tyrosine kinase inhibitor (TKI), was known to inhibit c-kit as well as BCR-ABL fusion protein, the target of chronic myeloid leukemia (CML). The first case report of a patient with GIST treated by imatinib showed an apparent clinical response and tolerable toxicity, which accelerated the clinical trials of imatinib for GIST patients [14]. In an early-phase clinical trial, imatinib treatment resulted in both a high rate of response by recurrent/metastatic GIST patients and controllable adverse events, and subsequent randomized clinical trials confirmed the clinical benefits of extending the survival of GIST patients by imatinib treatment; in these clinical trials, median OS of recurrent/metastatic GIST patients reached nearly to 5 years [15,16,17].

For surgically resectable GIST patients too, a randomized trial showed that imatinib treatment resulted in an improvement of progression-free survival (PFS) in the adjuvant setting [18]. A comparison of different imatinib treatment periods (three years vs. one year) showed that the longer treatment resulted in longer PFS; now, the 3-year continuation of imatinib in adjuvant settings is now recommended [3,19]. The survival benefit of an even longer continuation of adjuvant imatinib is being evaluated [20].

Based on sub-analyses of those clinical trials, predictive factors of imatinib resistance have been unveiled. Most major KIT mutations occur in exon 11, and this mutation is favorable for imatinib therapy; however, patients with a KIT mutation in exon 9, which accounts for 10–20% of GIST cases, were poor responders to imatinib therapy [21,22]. For GIST patients with exon 9 mutation, dose escalation of imatinib up to 800 mg would be the beneficial option [21].

### 2.2. Next to Imatinib: Clinical Trials of New TKIs for GISTs

Clinical trials of several new TKIs have been conducted for GIST patients resistant to imatinib, and as a result, the new TKIs sunitinib and regorafenib were approved. Sunitinib showed significant prolongation of PFS to placebo for imatinib-resistant GIST patients in a randomized clinical trial (27.3 weeks versus 6.4 weeks; *p* < 0.0001) [23]. Regorafenib was a TKI which extended PFS for GIST patients who were resistant to both imatinib and sunitinib; in phase III trial (GRID), median PFS of regorafenib was 4.8 months compared to 0.9 months of placebo (*p* < 0.0001) [24]. The new TKIs are known to inhibit multiple tyrosine kinases in addition to c-kit, such as vascular endothelial growth factor receptor (VEGFR), platelet-derived growth factor (PDGFR), fibroblast growth factor receptor (FGFR), and more. Of them, PDGFR has been known as the main mutation of GIST along with c-kit [13]. Therefore, the anti-PDGFR-specific agents crenolanib and olaratumab were tested as treatments for patients with imatinib-resistant GIST, mainly those with PDGFR mutation [25,26]. Second- or third-generation TKIs that are approved for treating CML such as dasatinib, nilotinib and ponatinib have also been examined as treatment for GIST, but the targets of those TKIs focus on BCR-ABL and its related mutations, specific targets of CML, and the patient responses in clinical trials have been modest [27,28].

## 3. Molecular Targeting Therapy for Non-GIST Soft Tissue Sarcoma (STS)

### 3.1. Pazopanib: First Targeting Therapy for Non-GIST STS

The developments of molecular targeted therapy for non-GIST soft tissue sarcomas (STSs) lagged behind those for GISTs by about 10 years; the main reasons for this lag are the diversity of the heterogeneity of STSs and the lack of driver mutations such as c-kit in GISTs. Though there were some patients who responded to cytotoxic agents and/or successfully treated by salvage curative surgeries, median OS of non-GIST STS patients remains less than two years [29]. However, the investigations of sarcoma genomics and mutations of signaling pathways have indicated several candidates for targeted therapy for non-GIST STSs, and the angiogenetic pathway was revealed to be one of the promising targets, as in many solid tumors [5,30,31].

Pazopanib is an oral anti-angiogenic drug that inhibits VEGFR, PDGFR, FGFR, c-kit and many other tyrosine kinases [32,33]. It is also approved for the treatment of renal cell carcinomas [34].

Based on the results of phase I trials in which six sarcoma patients out of 63 solid malignant tumor patients participated, the tolerability and recommended dose of pazopanib were evaluated [35]. In the phase II study EORTC 62043, soft tissue sarcomas patients were enrolled as four cohorts divided by their pathological diagnoses: leiomyosarcoma, synovial sarcoma, liposarcoma, and other histologies [36]. The primary end point was the progression-free rate at 12 weeks, and the outcomes were evaluated in each cohort; 18 of 41 (44%) patients in leiomyosarcoma cohort, 18 of 37 (49%) patients in synovial sarcoma cohort, 16 of 41 (39%) patients in other histologies cohort reached the progression-free at 12 weeks. On the other hand, accrual for liposarcoma cohort was stopped because of only three of the first 17 patients met progression-free at 12 weeks; with the central histopathologic reviews, however, two other patients who showed the progression-free at 12 weeks added to the liposarcoma cohort, so in the final results, five of 19 (26%) patients in liposarcoma cohorts reached the progression-free at 12 weeks. As a result, the STS without liposarcoma patients were enrolled in a phase III study (PALETTE). The median PFS was 4.6 months for the pazopanib-treated patients compared to 1.6 months for the placebo-treated patients (*p* < 0.0001), and the results of the PALETTE study were the foundation of the approval of pazopanib for STSs, as the first molecular targeted therapy for STS [37].

Liposarcoma patients were excluded from the PALETTE study based on the provisional results of the EORTC 62043 phase II study. However, in the final results of the EORTC study, the primary end point was also met in the liposarcoma cohort. As a result, some countries as Japan approved pazopanib for STSs including liposarcoma, and objective responses to liposarcoma were observed and reported in clinical practices [38,39]. Toward the approval of pazopanib for liposarcomas, preclinical investigations and prospective clinical trials for liposarcomas were conducted, and the results suggested that pazopanib has potential antitumor activities against liposarcoma, especially dedifferentiated liposarcoma; the phase II study of pazopanib for liposarcoma, in which well differentiated liposarcoma was excluded, showed 68.3% progression-free rate at 12 weeks and median PFS of 4.4 months [40,41].

A clinical biomarker of the response to pazopanib treatment for STS has not been established, but some predictive markers such as performance status, hemoglobin, and pathological grade were suggested based on the collected data from EORTC clinical trials [42]. The relationship of TP53 mutational status and response to pazopanib was evaluated in a small study; the TP53 mutant STS patients showed longer PFS, but a verification of that study’s results in a large-scale cohort has not been conducted [43].

### 3.2. Emerging Targeting Therapies for STS—New TKIs, New Antibodies, New Targets

After the introduction of pazopanib, many clinical trials have been performed for other antiangiogenic TKIs as treatments for non-GIST STSs: sorafenib, sunitinib, regorafenib, and more.

The TKIs sorafenib and sunitinib showed clinical benefits against many solid tumors approved prior to pazopanib, but the clinical trials of these agents as STS treatment are limited to single-arm phase II trials [44,45,46]. Regorafenib was already approved as a GIST treatment, as stated above [24], and a randomized phase II trial (REGOSARC) was also performed for non-GIST STSs: like the EORTC 62043 study of pazopanib, the response and survival benefit of regorafenib were evaluated in four cohorts (leiomyosarcoma, synovial sarcoma, liposarcoma, other histologies), and in each cohort, a control arm using a placebo was examined. The three cohorts other than the liposarcoma cohort showed PFS prolongation compared to the placebo arm [47]. The REGOSARC trial also showed the benefits of quality-adjusted survival [48]. However, the survival benefits of regorafenib in the REGOSARC trial were not very different from those of pazopanib; the median PFS of the regorafenib-treated patients in the non-liposarcoma cohorts was limited to four months and the overall survival to 13.4 months; moreover, regorafenib treatment for liposarcoma failed to result in PFS prolongation as pazopanib did.

Focusing on some particular histologies, it is possible that TKIs could achieve high responses. There are several case series of patients with alveolar soft part sarcoma (ASPS) in whom a high objective response rate (ORR) to sunitinib was obtained [49,50,51]. ASPS is known to be resistant to cytotoxic chemotherapy [52]. Subsequent to those case series, a clinical trial of ASPS patients treated with cediranib (known as a potent VEGFR inhibitor) was performed; in the phase 2 trial, 46 ASPS patients were enrolled and of them, 15 patients (35%) had a partial response [53]. This was a clearly higher ORR than those of other TKIs and even those of cediranib in other STS histologies [54].

The differences in the half maximal inhibitory concentration (IC_50_) of each TKI [33,55,56,57,58,59] are shown in Table 1. The inhibitory effects of each tyrosine kinase differ among the TKIs, but the critical points of their differences related to responses to STS are not yet clear.

Many more TKIs are now being investigated as STS treatment, such as anlotinib from China; though detailed function profiles of anlotinib were not proven, their responses for STSs are comparative to other TKIs (ORR 11.45%, median PFS 5.63 months) [60,61]. The appropriate selection and use of TKIs remain to be established.

In addition to TKIs, monoclonal antibodies have also been approved for STS. As is true of GISTs, PDGFR overexpression is observed in STSs, and this overexpression is related to poor prognoses [62,63]. As noted above, many TKIs can inhibit PDGFR, and the PDGFR-focused targeted drug olaratumab, a monoclonal antibody, has been approved for treating STSs.

In phase I clinical trials for solid tumors, olaratumab monotherapy provided 5.6 months of disease control in leiomyosarcoma patients [64]. For more intensive disease control, a combination of olaratumab and doxorubicin (the standard treatment agent for STS) was investigated, and a phase Ib/randomized phase II trial showed a higher ORR and the prolongation of overall survival by the olaratumab/doxorubicin combination compared to doxorubicin alone; 26.5 months for the olaratumab/doxorubicin combination versus 14.7 months for doxorubicin alone (*p* = 0.0003) [65]. Superiority to doxorubicin in OS is a milestone many other clinical trials and drugs had failed to get over. Based on those results, the U.S. Food and Drug Administration (FDA) approved olaratumab as breakthrough therapy, and the European Medicines Agency (EMA) also approved olaratumab before the completion of a phase III trial.

Though categorized as parts of traditional cytotoxic drugs, other new agents, trabectedin and eribulin, were also approved for STS treatments. Trabectedin is a marine-derived compound isolated from *Ecteinascidia turbinata*, which binds a guanine residue in the DNA minor groove and alters DNA interactions with transcription factors. Trabectedin was first approved in Europe based on the phase 2 trial, and the clinical data from practices showed that the clinical benefit of trabectedin was observed especially in l-sarcoma—leiomyosarcoma and liposarcoma [66,67]. That was the reason why the phase 3 trial of trabectedin was performed for leiomyosarcoma and liposarcoma. In the phase 3 trial, the median PFS of trabectedin arm was 4.2 months, which was significantly longer than those of control arm, dacarbazine (1.5 months, *p* < 0.001); however, there were no significant differences in the median OS (12.4 months in trabectedin arm and 12.9 months in dacarbazine arm; *p* = 0.37) [68]. In contrast to trabectedin, eribulin showed OS improvement without PFS prolongation. Eribulin was similar to trabectedin in some points; which is also marine-derived compound (*Halichondria okadai*) and shows clinical benefit to l-sarcomas [69,70]. The subject of phase 3 trial of eribulin was limited to l-sarcoma same as trabectedin; the result, as described before, showed the OS prolongation of eribulin arm compare to dacarbazine (13.5 months vs. 11.5 months, *p* = 0.0169), but not the PFS improvement (2.6 months vs. 2.6 months; *p* = 0.23) [71]. By the subanalysis of the phase 3 trial the PFS and OS prolongation of eribulin were observed in liposarcoma, but not in leiomyosarcoma; thus, FDA approved eribulin only to liposarcoma, though in some countries such as Japan eribulin is approved to STS other than liposarcoma [72,73].

Molecules of intracellular signaling pathways are also potential targets for new drugs; these include phosphatidylinositol 3-kinase (PI3K)/AKT/mammalian target of rapamycin (mTOR), and intranuclear mouse double minute 2 homolog (MDM2) and cyclin-dependent kinase (CDK)4/6 [5]. Nuclear export compound of oncogenic proteins and epigenetic regulation systems by histone deacetylases (HDACs) might be a treatment target for STS based on preclinical data.

Regarding the PI3K/AKT/mTOR pathway, the mTOR inhibitor ridaforolimus was investigated mainly for sarcoma; a phase 3 trial of ridaforolimus showed the prolongation of PFS in sarcoma patients (mainly STS patients, but some bone sarcoma patients were included in this study) in the maintenance setting after chemotherapy, but the clinical benefit was too small to result in approval [74]. In only perivascular epithelioid cell tumors (PEComas), which are known to be linked through activation of the mTOR pathway, a high rate of response to the mTOR inhibitor sirolimus was observed, and small case series and case reports have been reported [75]. MDM2 and CDK4/6 are highly amplified in well-differentiated/dedifferentiated liposarcoma [76,77]. The CDK4/6 inhibitor palbociclib is approved for treating hormone-receptor-positive breast cancer [78]. Palbociclib also showed modest disease control effects in liposarcoma, but the clinical evidence is limited to phase II trials [79,80]. The evidence regarding MDM2 inhibitors as a treatment for liposarcoma is limited to preclinical data and a phase I trial [81,82], but the double inhibition by MDM2 and CDK4 might be synergic, which would be worth evaluating [83]. In a preclinical study, an HDAC inhibitor showed the potential to be effective against sarcoma cell lines [84], but the clinical evidence shown by monotherapy or combinations is limited to phase I and II trials [85,86,87]. As for a nuclear export inhibitor, based on the promising result of the preclinical trial, in which XPO1 inhibitor selinexor showed antitumor effects in sarcoma cell line, a clinical trial of selinexor has just started; in a recent phase Ib trial, its disease control against liposarcoma is especially noted [88,89].

Approximately 20–25% of STS patients have chromosomal translocations [3]. These translocations have had an important role in the diagnosis of STS subtypes, but there had been no targeted therapies that focus on the translocation or fusion proteins of STS until recently. Trabectedin, already described as an cytotoxicagent to l-sarcoma, has been demonstrated to modulate the transcription of the oncogenic fusion proteins, FUS-CHOP of myxoid/round cell liposarcoma in particular, and translocation-related sarcoma were shown to respond to trabectedin in clinical practice [90,91].

More therapies targeted to specific fusion gene/proteins have been emerging; anaplastic lymphoma kinase (ALK) is well known as a treatment target of non-small cell lung cancer, and ALK-related fusion gene is also observed in an paricular STS, i.e., inflammatory myofibroblastic tumor (IMT). From the early period of the development of ALK-targeted therapy, there were case reports of IMT patients who responded to an ALK inhibitor, crizotinib [92]. Because of their rarity, prospective clinical trials of ALK inhibitors as treatment for ALK-arranged sarcomas have been difficult to perform, but due to the Children’s Oncology Group’s perseverance, phase I and II crizotinib trials were completed and high responses with good prognoses were certified for pediatric IMT patients; in the phase II trial, complete response rate was observed in five of 14 (36%) patients [93,94]. Rhabdomyosarcoma is also known to show ALK aberrations, but these are different from the translocation; in a preclinical trial an ALK inhibitor seemed to be inactive against rhabdomyosarcoma [95,96]. Instead, PAX3-FOXO1 translocation, an indicator of poor prognosis of alveolar rhabdomyosarcoma, is considered to be a treatment target; in preclinical study, PAX3-FOXO1 requires the BET bromodomain protein BRD4 to function at super enhances, so BRD4 inhibitor is suggested to be a new targeted drug for alveolar rhabdomyosarcoma [97,98]. Translocation related to tropomysin receptor kinase (TRK) was recently detected in broad malignancies at low frequency, but this appears to be very promising treatment target [99,100]. In fact, treatment with the TRK inhibitor LOXO-101 (larotrectinib) resulted in promising responses by STS patients with TRK fusion [101,102].

To date, clinical trials of STS have tended to enroll patients with specific histologies or targets rather than all or nearly all STS histologies, as is the case for pazopanib or olaratumab described above. The fact mTOR inhibitor ridaforolimus failed to be approved after the randomized clinical trials for all sarcomas, though that the drug responses to a particularly effective subtype (PEComa) was an also lesson. Recently new clinical trials of new targeted therapies for STSs, such as CDK4 inhibitors and a nuclear export inhibitor, the subtypes of STS targeted could be narrowed down from early phase of clinical trials.

## 4. Molecular Targeted Therapy for Bone Sarcoma

The introduction of systemic chemotherapy has improved the outcomes of bone sarcoma patients, mainly as neoadjuvant/adjuvant therapy; methotrexate, cisplatin and doxorubicin combination chemotherapy for osteosarcoma, and alternating chemotherapy of vincristine, doxorubicin, cyclophosphamide and ifosfamide, etoposide for Ewing sarcoma [103,104]. These benefits were brought from traditional cytotoxic agents, however, and for recurrent/metastatic bone sarcoma patients, salvage chemotherapy has produced only poor responses. Many clinical trials of new targeted drugs have been conducted, but none of the investigated agents showed survival benefits [105].

Single angiogenic agents or their combinations have been revealed to be inactive in clinical trials of bone sarcomas [106,107,108], and many potential mutations which are meaningful targets in other malignancies did not work in bone sarcoma; human epidermal growth factor 2 (HER2) is a representative example. HER2-targeted therapies are now essential components of breast cancer and gastric cancer treatments, but HER2-targeted therapy did not show any clinical benefit for bone sarcomas [109]. Insulin-like growth factor 1 receptor (IGF-1R) was a candidate as a treatment target of Ewing sarcoma, and early-phase clinical trials of IGF-1F inhibitors, especially of figitumumab, showed a modest response among recurrent/metastatic Ewing sarcoma patients, including complete remission [110,111,112]. However, further clinical trials of figitumumab were discontinued after the negative results of a phase III trial in non-small cell lung cancer [113].

Patients with giant-cell tumors of the bone showed clinical responses to a receptor activator of nuclear factor kappa-B ligand (RANKL) inhibitor and RANKL inhibitor; denosumab is approved now [114]. However, clinical evidence of effectiveness of RANKL inhibitors against osteosarcoma or other high-grade malignant bone sarcomas are lacking, even though RANKL expression in bone sarcoma was observed and an antitumor effect in a preclinical model was reported [115,116,117]. The clinical effects of bone-modifying agents must be evaluated cautiously; for example, a randomized clinical trial examining zoledronic acid treatment for osteosarcoma showed no survival benefits [118].

New molecular targeted drugs for bone and soft tissue sarcomas described are summarized in Table 2 below.

## 5. Immunotherapy for Bone and Soft Tissue Sarcoma

In the 2010s, immune-checkpoint inhibitors emerged in the field of oncology and immediately became new standard therapies for many malignancies [119]. The expression of ligand of programmed death-1 (PD-1; i.e., PD-L1) and/or PD-L2 was considered as one of the most important biomarkers of PD-1 inhibitors; a high expression of PD-L1 could be a predictive factor of response to anti-PD-1 therapy, and in some malignancies such as non-small cell lung cancers, the evaluation of PD-L1 expression was inseparably linked to the indication of immunotherapy [120,121].

The expression of PD-L1 in bone and soft tissue sarcomas has been evaluated, and the PD-L1 expression was as high as that in other malignancies in which anti-PD-1/PD-L1 therapies showed clinical evidence of benefit [122,123,124,125]. However, in prospective clinical trials, anti-PD-1 therapy for bone and soft tissue sarcomas resulted in minimal patient responses [126,127,128,129]. In a phase 2 trial of pembrolizumab (SARC028), only seven of 40 (18%) STS patients and two of 40 (5%) bone sarcoma patients showed objective clinical responses; the reason of dissociation of PD-L1 expression and clinical responses in sarcomas are not clear. In SARC028, undifferentiated pleomorphic sarcoma (UPS) patients showed a high response rate to anti-PD-1 therapy; 4 of 10 (40%) patients responded to pembrolizumab, which might be due to high mutation burden in UPS [130]. In fact, however, there are also some case reports of STS patients with histologies other than UPS who responded to anti-PD-1 therapy [131,132]. A report from clinical trials suggests that the use of PD-1 targeted therapy might bring about the activation of indoleamine 2,3-dioxygenase 1 (IDO1), which could be a new target of combination immunotherapy [133].

Immunotherapy with chimeric antigen receptor-modified T cells (CART) or dendritic cells is also being investigated [134,135]. Targeted immunotherapy with the cancer-testis antigen NY-ESO-1 for synovial sarcoma has shown especially promising results, with the objective response to 11 of 18 (61%) patients; a limitation is that the indication for this immunotherapy is limited to patients with a specific human leukocyte antigen (HLA) haplotype, HLA-A*0201 [136], but to selected patients, CART would be a powerful treatment option.

## 6. Future of Targeted Therapy for Sarcomas: New Clinical Trial Designs Adapted to Sarcomas

As a result of the development of treatment targets in preclinical trials, the candidate cancers from each clinical trial have narrowed from whole bone and/or soft tissue sarcomas to specific histologies or sarcomas with specific mutations. It would be of great benefit for patients to avoid receiving futile treatments, but for rare diseases such as bone and soft tissue sarcomas, a sufficient patient enrollment and even the planning of clinical trials would be more difficult based on the traditional clinical trial design for the evaluations of data. Performing clinical trials for these ‘ultrarare’ disease groups is challenging for the pharmaceutical industry, too. Both positive and negative results of clinical trials for other major malignancies might bring discontinuation the investigations of drugs for rare diseases, like CDK4 inhibitor palbociclib and IGF-1R inhibitor figitumumab as stated above.

A new paradigm for clinical evidence emerged recently, precision medicine [6]. Based on precision medicine, regardless of the amount of disease, therapies targeted to individual patients’ own mutation or target can be performed. The design of clinical trials can also be arranged to work within this paradigm, from large-scale randomized clinical trials to basket and umbrella trials [7]. A basket trial evaluates multiple diseases with a single target or mutation for specific targeted treatment, and an umbrella trial evaluates various subgroups within a single disease for multiple targeted therapies fitted to their own target. Of them, for sarcoma patients, enrollment in a basket trial according to their targets is ideal. The clinical trial of the TRK inhibitor larotrectinib is an excellent example, in which clinical responses including complete remission were observed in sarcoma patients with TRK fusions [101,102]. As for clinical trials in progress, CREATE trial (EORTC 90101), which evaluates the clinical effects of crizotinib for rare sarcomas classified by MET mutation, is designed on a similar concept [137,138].

For appropriate enrollments of bone and soft sarcoma patients to clinical trials of targeted drugs, the importance of accurate pathological diagnoses continues to increase. In sarcoma, discordances of pathological diagnoses between different pathologists are not rare; there is a report that more than 40% of pathological diagnoses changed by the review of a second opinion [139]. Many reasons obstruct accurate, concordant diagnoses; complexity and diversity of sarcoma diagnoses, insufficiency of specimens, and so on. Even heterogeneity of tumors in one patients might be often observed in sarcomas. Investigation of methods of diagnoses which have less discordances to evaluate are in need, for both accurate diagnoses and detecting treatment targets.

In addition, for the appropriate enrollment of rare-disease patients (such as those with bone and soft tissue sarcomas) in clinical trials using this new paradigm, social systems must increase their ability to quickly make use of the products of preclinical and clinical trials. One example of new coping with the social system is NETSARC: the French National Cancer Institute funded a network of 26 reference sarcoma centers with specialized multidisciplinary tumor board. Five years after the construction of NETSARC, it is reported that the compliance with clinical practice guidelines and actual prognoses of sarcoma patients were getting better [8]. Nationwide networks and centralization could improve prognoses of patients at present, more than improving the accrual of clinical trials. The construction of a global registry of rare diseases would contribute significantly to future investigations in this field.

Incorporation of adolescent and young adult (AYA) patients, which usually indicates patients of 15–39 years old, to clinical trials is also an important problem for the developments of clinical trials to bone and soft tissue sarcomas; incidence of bone sarcomas peaks in late teens and about 20% of all STS are diagnosed at AYA ages, and some histological types of sarcoma like dermatofibrosarcoma protuberans appear most frequently in this population [140]. In general, prognoses of AYA cancer patients are not inferior to those of children and older adults, but their improvement has been slower than those of other ages [141]. One reason for the stagnation of improvement of AYA cancer patients’ outcomes would be a low rate of enrollments in clinical trials; from pediatric to AYA ages, accrual rates of clinical trials rapidly fall, and is even referred to as “AYA cliff” [140,142]. In fact, there might be disparities in access to cancer centers for AYA cancer patients [143]. As pediatric oncologists have been successful in keeping high rates of clinical trials for pediatric cancer patients, cooperation with pediatric oncologists and medical oncologists who see adult patients is necessary to improve quantity and quality of registry and clinical trials for AYA sarcoma patients. In addition to the diseases themselves, many AYA cancer patients have social problems, such as issues of employment, education and financial stability [144]; so, constructions of social support for these patients are urgent.

## Figures and Tables

**Table 1:** Differences in the IC_50_ of TKIs tried or approved to treat STS (μmol/L).

Tyrosine Kinase	Pazopanib [33]	Sorafenib [55,56]	Sunitinib [56,57]	Cediranib [56]	Regorafenib [56,57,58,59]
VEGFR-1	0.01	-	0.002	0.005	0.013
VEGFR-2	0.03	0.09	0.009	<0.001	0.0042
VEGFR-3	0.047	0.02	0.017	<0.003	0.046
PDGFR-α	0.071	-	0.069	-	0.136
PDGFR-β	0.084	0.057	0.002	0.005	0.022
c-Kit	0.074	0.068	0.022	0.002	0.007
FGFR-1	0.14	0.58	0.88	-	0.202
FGFR-3	0.13	-	-	-	-
FGFR-4	0.8	-	-	-	-
c-fms	0.146	-	-	-	-
LCK	0.411	-	-	-	-
ITK	0.43	-	-	-	-
FAK	0.8	-	-	-	-
p38α	1.056	-	-	-	-
Abl1	2	-	-	-	-
JNK1	2.466	-	-	-	-
Ret	2.8	-	-	-	0.0015
Src	3.09	-	-	-	-
GSK3	3.46	-	-	-	-
JNK3	4.065	-	-	-	-
ALK6	4.266	-	-	-	-
Tie-2	4.52	-	-	-	-
Met	6	-	-	-	-
IGF-1R	8	-	-	-	-
CSF-1R	-	-	0.05–0.10	-	-
JNK2	10.233	-	-	-	-
Flt-3	>20	0.058	0.25	>1	-
Raf	-	0.006	-	-	-

**Table 2:** Current targeted therapies to bone and soft tissue sarcomas.

Drug	Molecular Targets	Pathology	Phase	Clinical Outcomes	References
anlotinib	c-kit, FGFR1-4, PDGFR α/β, Ret, VEGFR2/3	non-GIST STS	P2	ORR 11.45%, PFS 5.63 months	[60,61]
cediranib	VEGFR1-3	ASPS	P2	ORR 35%, disease control rates 84% at 24 weeks	[53]
crizotinib	ALK	IMT	P2	ORR 86% (36% of complete remission), median duration of treatment 1.63 years	[94]
denosumab	RANKL	Giant cell tumor of bone	approved	Tumor response * 86%	[114]
figitumumab	IGF-1R	Ewing sarcoma, osteosarcoma	P2	ORR 14.2%, OS 8.9 months	[112]
imatinib	BCR-ABL, c-kit, PDGFR	GIST	approved	For recurrent/metastatic: - PFS 18 months, OS 55 months In the adjuvant setting: - 1-year continuation: 98% of 1-year recurrence free survival; 47.9% of 5-year recurrence free survival - 3-year continuation: 65.6% of 5-years recurrence free survival	[15,16,17,18,19]
larotrectinib	TRK fusion	STS with TRK fusion	P1	Some cases with drastic responses are reported	[101,102]
nilotinib	BCR-ABL, c-kit, PDGFR	GIST	P3	Two-year PFS 51.6% (significantly lower than 59.2% in imatinib)	[27]
olaratumab	PDGFR α	non-GIST STS, particularly leiomyosarcoma	P1-2	ORR 18.2% **, PFS 6.6 months **, OS 26.5 months **	[65]
		GIST	P2	PFS 32.1 weeks (PDGFR α mutant) PFS 6.1 weeks (PDGFR α wild type)	[26]
palbociclib	CDK4/6	liposarcoma	P2	PFS 17.9 weeks	[79,80]
pazopanib	c-kit, FGFR, PDGFR, VEGFR	non-GIST STS other than liposarcoma	approved	ORR 9%, PFS 4.6 months, OS 12.5 months	[37]
		liposarcoma	P2	PFS 4.4 months, OS 12.6 months	[41]
regorafenib	c-kit, FGFR, PDGFR, VEGFR	GIST	approved	ORR 4.5%, PFS 4.8 months	[24]
		non-GIST STS	P2	Leiomyosarcoma: PFS 3.7 months liposarcoma: PFS 1.1 months synovial sarcoma: 5.6 months other sarcoma: 2.9 months	[47]
ridaforolimus	mTOR	bone sarcoma and STS	P3	PFS 17.7 weeks (maintenance after chemotherapy)	[74]
selinexor	XPO1	non-GIST STS, particulary liposarcoma	P1	ORR 0%, stable disease ≥ 4 months in 33%	[89]
sorafenib	c-kit, FGFR, PDGFR, Ret, VEGFR	non-GIST STS	P2	angio/vascular sarcoma: PFS 5.6 months, OS 12.2 months leiomyosarcoma: PFS 5.2 months, OS 12.5 months other sarcoma: PFS 2.8 months, OS 10.1 months	[45]
		osteosarcoma	P2	PFS 4 months, OS 7 months	[106]
Sunitinib	c-kit, FGFR, PDGFR, VEGFR	GIST	approved	ORR 7%, PFS 27.3 weeks	[23]
		non-GIST STS	P2	Liposarcoma: PFS 3.9 months, OS 18.6 months Leiomyosarcoma: PFS 4.2 months, OS 10.1 months Malignant fibrous histiocytoma: PFS 2.5 months, OS 13.6 months	[46]
Trastuzumab	HER2	osteosarcoma	P2	30 months event free survival rates 32% ***, 30 months OS 59% ***	[109]
vorinostat	HDAC	non-GIST STS	P2	ORR 0%, PFS 3.2 months, PS 12.3 months	[86]

* defined as elimination of at least 90% of giant cells or no radiological progression of the target lesion up to week 25; ** results by combination with doxorubicin; *** results by combination with chemotherapy.

## Abbreviations

ALK	anaplastic lymphoma kinase
ASPS	alveolar soft part sarcoma
AYA	adolescent and young adult
CART	chimeric antigen receptor T cell
CDK	cyclin-dependent kinase
CML	chronic myeloid leukemia
FGFR	fibroblast growth factor receptor
GIST	gastrointestinal stromal tumor
HDAC	histone deacetylases
HER2	human epidermal growth factor 2
HLA	human leukocyte antigen
IC_50_	half maximal inhibitory concentration
IDO1	indoleamine 2,3-dioxygenase 1
IGF-1R	insulin-like growth factor 1 receptor
IMT	inflammatory myofibroblastic tumor
mTOR	mammalian target of rapamycin
ORR	objective response rate
OS	overall survival
PD-1	programmed death-1
PDGFR	platelet-derived growth factor receptor
PEComa	perivascular epithelioid cell tumor
PFS	progression-free survival
PI3K	phosphatidylinositol 3-kinase
RANKL	receptor activator of nuclear factor κB ligand
STS	soft tissue sarcoma
TKI	tyrosine kinase inhibitor
TRK	tropomysin receptor kinase
UPS	undifferentiated pleomorphic sarcoma
VEGFR	vascular endothelial growth factor receptor
